# Spin-Dependent Transport in Fe/GaAs(100)/Fe Vertical Spin-Valves

**DOI:** 10.1038/srep29845

**Published:** 2016-07-19

**Authors:** P. K. Johnny Wong, Wen Zhang, Jing Wu, Iain G. Will, Yongbing Xu, Ke Xia, Stuart N. Holmes, Ian Farrer, Harvey E. Beere, Dave A. Ritchie

**Affiliations:** 1 York-Nanjing Joint Center in Spintronics and NanoEngineering, School of Electronics Science and Engineering, Nanjing University, Nanjing 210093, China; 2 Spintronics and Nanodevice Laboratory, Department of Electronics, University of York, YO10 5DD, UK; 3 Department of Physics, University of York, YO10 5DD, UK; 4 Department of Physics, Beijing Normal University, Beijing, China; 5 Toshiba Research Europe Limited, Cambridge Research Laboratory, 260 Cambridge Science Park, Milton Road, Cambridge, CB4 0WE, UK; 6 Department of Electronic and Electrical Engineering, University of Sheffield, Mappin Street, Sheffield, S1 3JD, UK; 7 Cavendish Laboratory, University of Cambridge, Madingley Road, Cambridge, CB3 0HE, UK

## Abstract

The integration of magnetic materials with semiconductors will lead to the development of the next spintronics devices such as spin field effect transistor (SFET), which is capable of both data storage and processing. While the fabrication and transport studies of lateral SFET have attracted greatly attentions, there are only few studies of vertical devices, which may offer the opportunity for the future three-dimensional integration. Here, we provide evidence of two-terminal electrical spin injection and detection in Fe/GaAs/Fe vertical spin-valves (SVs) with the GaAs layer of 50 nanometers thick and top and bottom Fe electrodes deposited by molecular beam epitaxy. The spin-valve effect, which corresponds to the individual switching of the top and bottom Fe layers, is bias dependent and observed up to 20 K. We propose that the strongly bias- and temperature-dependent MR is associated with spin transport at the interfacial Fe/GaAs Schottky contacts and in the GaAs membranes, where balance between the barrier profiles as well as the dwell time to spin lifetime ratio are crucial factors for determining the device operations. The demonstration of the fabrication and spin injection in the vertical SV with a semiconductor interlayer is expected to open a new avenue in exploring the SFET.

Semiconductor (SC) spintronics is a promising field, holding great promises for non-volatility, power amplification, and low heat dissipation in devices that combine data storage and processing^1^,^2^,^3^. Over the past couple of years, this field has witnessed a number of major breakthrough in demonstrating electrical injection and detection, and manipulation of charge carrier spins in several technologically important electronic materials, including conventional SCs^4^,^5^,^6^,^7^,^8^,^9^,^10^,^11^, organic molecular materials^12^,^13^,^14^, and also the recently discovered two-dimensional layered materials^15^,^16^,^17^,^18^,^19^,^20^. While the latter two types of materials can serve as excellent platforms for the exploration of potential spintronic applications that concern with chemical tunability, low-dimensionality, and so on, conventional SCs remain the most established candidates for spintronics, not only because of their well-known material properties, but also due to their overwhelming roles in modern electronic industry. In fact, many of the abovementioned breakthrough studies were achieved using conventional SCs^4^,^5^,^6^,^10^,^11^.

Experimental observations of all-electrical spin injection/detection and transport in SCs have mainly been demonstrated in lateral devices, where the injected spin-polarized current flows parallel to the device surface^4^,^7^,^9^,^11^. Such devices are structurally similar to the spin transistor proposed by Datta and Das^21^, and usually exhibit multiple terminals arranged in the so-called non-local geometry^4^,^7^,^11^,^15^,^19^. Yet if spintronics is to revolutionize the mainstream SC technology, two-terminal devices should instead be closer to the realm of practical applications. Indeed, magnetoresistance (MR) effect due to diffusive electron transport has been predicted to exist in a two-terminal spin-valve structure, with a non-magnetic SC spacer layer^22^. Rarely achieved in practice though^12^, such a proposal may be realized in a vertical structure in which the SC spacer is sandwiched by two ferromagnetic (FM) electrodes^14^,^16^,^20^,^23^. Here this configuration is referred to as the current-perpendicular-to-plane (CPP) geometry, since the current flow is perpendicular to the device plane. In terms of the device fabrication, one practical challenge might however lie on the precise control of the SC layer thickness. Depending on both applications and fundamental parameters of the target SC, such as spin-orbit coupling and thus spin lifetime, this may span widely from sub-micrometers down to a few nanometers. Another main challenge is expected to concern with the robustness and engineering of the FM/SC interfaces involved for high efficient spin injection and detection.

In this Report, we present the fabrication and characterizations of two-terminal vertical Fe/*n*-doped GaAs(100)/Fe spin-valve (SV) devices. An array of techniques has been developed by combining *ex-situ* selective chemical etching of GaAs/AlGaAs/*n*-GaAs epilayers and *in-situ* deposition of both top and bottom Fe electrodes in the same growth runs by molecular beam epitaxy (MBE). In this study, the thinnest GaAs membranes fabricated with a high yield are as thin as 50 nm. The best device thus fabricated exhibit SV signals up to 20 K with an MR value of ~0.5% at 5 K. Furthermore, the MR corresponds to the individual switching of the Fe electrodes and shows strong bias- and temperature dependence. These observations as a whole confirm spin-dependent transport in the two-terminal device.

## Results

**Figure 1:**
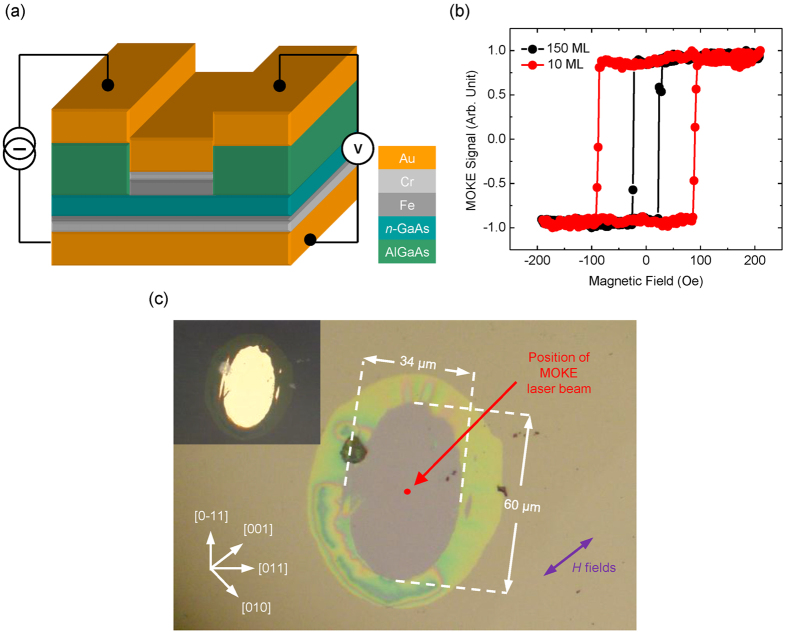
Two-terminal Fe/GaAs(100)/Fe vertical spin-valves and spatially-resolved magneto-optical Kerr effect measurements of Fe electrodes on etched GaAs membrane. (**a**) Schematic representation of the spin-valve (SV) devices with two Fe electrodes sandwiching a chemically etched GaAs(100) membrane; (**b**) Magnetic hysteresis loops of 150 ML and 10 ML thick Fe electrodes characterized by magneto-optical Kerr effect (MOKE) at room temperature. (**c**) Optical microscope image of a 50 nm thick GaAs membrane showing the position where the MOKE measurements were taken at and the crystallographic directions of the GaAs. The external magnetic fields, *H*, were applied in-plane along the [001] direction of the membrane for both MOKE and magnetoresistance (MR) measurements. Inset shows light illumination from an optical microscope transmitting through the thin membrane, prior to *in-situ* Fe depositions.

A schematic diagram of the SV devices is shown in Fig. 1(a). The devices were measured in the CPP geometry using a four-terminal setup, and the external magnetic fields needed to magnetize the Fe electrodes were applied in-plane (see Fig. 1(c)). In order to achieve a large difference in coercivity between the two FM electrodes, the thicknesses of the top and bottom Fe were intentionally chosen as 150 and 10 monolayer (ML), respectively. To ascertain this, we show in Fig. 1(b) the magnetic hysteresis loops of the two Fe layers obtained by magneto-optical Kerr effect (MOKE) at room temperature (RT). Besides having distinguishable coercive fields, the Fe electrodes exhibit both high squareness and sharp magnetization switching, which are highly essential for any MR measurements to be performed in practice. An optical microscope image of a representative 50 nm thick GaAs membrane along with its major crystallographic axes is illustrated in Fig. 1(c). After the etching process, the membrane attains an elliptical shape with a lateral size of about 46 × 80 μm^2^. The red dot marked in the figure indicates the position at which the spatially resolved MOKE measurements were taken. We notice that magnetic anisotropies exist in the FM layers, rendering dissimilar switching behaviors along different applied field azimuths with respect to the crystallographic directions of the GaAs membrane. Hereafter, for illustrative purposes, we shall mainly discuss our results obtained along the [001] direction, while those along other azimuths can be found in the Supplementary Information.

**Figure 2:**
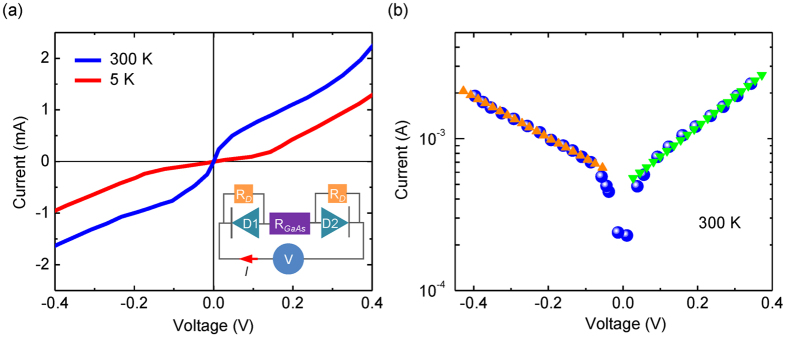
Electrical characterization of two-terminal Fe/GaAs/Fe SV device. (**a**) Four-point I–V characteristics of the SV device at 5 and 300 K, respectively. Inset shows a back-to-back diode circuit to account for the observed I–V responses. D1 refers to a forward-biased Fe/GaAs Schottky contact, whereas D2 a reverse-biased contact. The parallel resistors R_D_ represent conduction paths due to potential diode imperfections at the Fe/GaAs interfaces. (**b**) Logarithmic plot of the I–V curve at 300 K. The linear dependence of the current on both forward and reverse bias is numerically fitted.

The working principle of the two-terminal SV device is elucidated using a simple electrical circuit, as shown in the inset of Fig. 2(a). To take into account the Fe/*n*-GaAs Schottky barriers in the actual device, this circuit consists of two diodes, *D*_*1*_ and *D*_*2*_, each contributing a parallel resistance of *R*_*D*_ due to potential diode imperfections. Since the two energy barriers are always oppositely biased, the diodes are assigned to connect back-to-back in series with an additional resistance *R*_*GaAs*_, given by the GaAs membrane. Such a model has two important implications. First, at relatively low bias, the total voltage is expected to be distributed over the two Schottky barriers. Second, the device current is mainly limited by the leakage current of the reverse-biased Schottky barrier, *i.e*. spin injector, at one of the two Fe/GaAs interfaces.

As shown in Fig. 2(a), the actual device displays non-linear and asymmetric current–voltage (I–V) characteristics. The polarity in these measurements is such that forward bias corresponds to a positive voltage applied to the 150 ML Fe electrode. Here, the non-linearity is consistent with the expected non-Ohmic contacts between the membrane and the Fe electrodes, whereas the asymmetry in the I–V curves is associated with different barrier heights^24^. We also note that, for both bias polarities, the device current increases rapidly at relatively low voltages. Generally, there are two main mechanisms governing electron transport across an Schottky barrier^25^. The first is by quantum mechanical tunneling, which is possible only if the barrier width is sufficiently thin. The other is by thermionic emission, in which an electron is energetic enough to go over the barrier. In many cases, the second mechanism is thermally induced, and therefore strongly depends on temperature. To better understand the roles of these mechanisms in our device, we have estimated the depletion widths of the Fe/GaAs Schottky contacts, which are in the order of ~17 nm at zero bias^25^. While apparently too thick for tunneling, one has to realize that these values are referring to the bottom widths of the barrier energy landscapes. When applying a relatively small bias, electron tunneling via the narrower, higher energy part of the landscapes will become possible, thus giving rise to a device current. We have additionally analyzed the logarithmic plot of the RT I–V curve, as depicted in Fig. 2(b). The linear dependence of the current on both forward and reverse bias further reveals the predominant role played by the electron tunneling at both Schottky contacts. Using the thermionic-field emission model^25^, the device current *I* is linked to the Schottky barrier height *ϕ*_*b*_ and the tunneling parameter *E*_*0*_ as 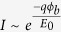, where the latter, which is related to the semiconductor properties, such as doping density, effective mass, and permittivity, can be extracted from the logarithmic plot in Fig. 2(b). The ratio of *k*_*B*_*T*/*E*_*0*_ may then serve as a measure of the relative contributions of thermally mediated and tunneling events. In the present study, it has been estimated that *E*_*0*_ is about twice the value of *k*_*B*_*T* at RT, thus evidencing the important role of electron tunneling in our devices. Upon cooling down to 5 K, the leakage current of the device is indeed reduced, thus reflecting that part of the device current measured at RT is thermally mediated as expected. On the other hand, via the abovementioned relationship, the barrier heights of the 10 and 150 ML Fe contacts have also been extracted as 0.77 and 0.80 eV, respectively.

**Figure 3:**
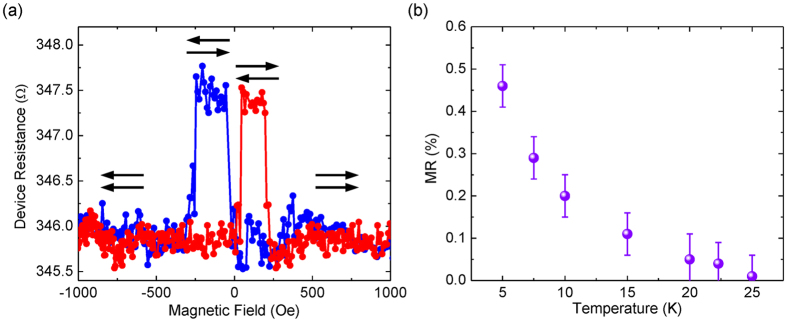
Magnetoresistance measurement of Fe/GaAs/Fe device at 5 K. The MR measurements were performed using a forward bias of 0.32 V with in-plane magnetic field along the [001] direction of the GaAs membrane. The arrows in the figure indicate the relative alignments of the magnetizations of the 150 ML and 10 ML Fe electrodes. (**b**) Temperature dependent MR for the device measured using the same bias as in (**a**).

The device MR measured at 5 K is shown in Fig. 3(a). As the magnetic field sweeps from −1000 to 1000 Oe, the magnetization alignment of the top and bottom Fe electrodes switches from parallel to antiparallel, and finally back to parallel, resulting in an observed resistance plateau. Here the MR is defined as *MR* = (*R*_*AP*_ − *R*_*P*_)/*R*_*P*_ where *R*_*P*_ and *R*_*AP*_ are respectively the device resistance at the parallel and antiparallel state. The MR value of the SV device is accordingly determined as 0.46% at 5 K. It is worth pointing out that the magnetic switching behavior here in the MR measurements is slightly different from that obtained by MOKE at RT. Such a disparity is mainly stemmed from the low measurement temperatures, at which the magnetization of the Fe films becomes harder to switch, possibly due to stronger magnetic anisotropies than at RT. In order to rule out the possibility that the observed MR signals are originated from spurious effects^26^,^27^,^28^, such as anisotropic magnetoresistance, Lorentz magnetoreistance, and magnetic stray fields of the Fe contacts, we have fabricated control devices with single Fe electrodes. None of these devices exhibits an apparent MR signal under the same experimental conditions, thus implying that the observed SV effect is stemmed from spin transport in the GaAs membrane. Figure 3(b) shows the temperature dependence of the MR signal. One sees that the MR drops off steeply as temperature rises, and only persists up to ~20 K. Such a rapid vanishment might be attributed to a combined effect involving a decreased spin lifetime^29^ and an enhanced thermionic emission over the Fe/GaAs Schottky barriers at higher temperatures^24^,^25^. However, considering that the thermal energy (*k*_*B*_*T*) changes in the temperature-dependent measurements, *i.e.* 5 K versus 20 K, is merely ~1.7 meV, the decrease in the spin lifetime of the electrons injected into the GaAs membrane seems to be a more reasonable cause of such MR decay. It is known that the spin relaxation in highly *n*-doped GaAs, as we have here, is primarily governed by the D’Yakonov and Perel’ type mechanism^29^. This results in a spin dephrasing rate of 1/τ_sf_ ∝ *T* ^3^, where τ_sf_ is spin lifetime and *T* is temperature.

**Figure 4:**
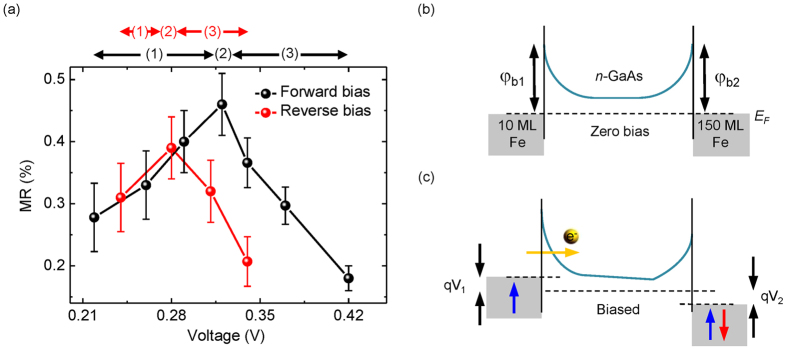
Bias dependence of MR signal. (**a**) Bias dependence of the device MR at 5 K for both forward and reverse bias; (**b**,**c**) Schematics of the device band diagrams under zero and low bias conditions. φ_b1_ and φ_b1_ are the respective Schottky barrier heights for the two Fe/GaAs contacts, whereas qV_1_ and qV_2_ refer to the band offsets of the contacts under a low bias. Operation of the two-terminal device depends on the subtle balance between the energy profiles of the back-to-back Fe/GaAs Schottky contacts. For an observable MR, spin-polarized electrons have to be injected into the GaAs membrane by tunneling via a reverse biased Schottky contact, and be detected at a forward biased contact by spin-filtering (*i.e.* electron tunneling). In case of relatively high biases in which strong band bending occurs, the injected spin-polarized electrons may surmount the detector barrier height, not contributing any spin-dependent signal in the device.

As extensively discussed in the literature, the conductivity mismatch between FM metals and SCs constitutes an intrinsic obstacle for efficient electrical spin injection^30^. Here, the highly doped Fe/GaAs Schottky-tunnel contacts in our SV device serve as spin-dependent resistive energy barriers, alleviating such a mismatch problem^22^,^31^,^32^. While for electrical spin detection, previous studies have revealed that tunneling electrons are responsible for spin-dependent transport across MBE-grown FM metals/GaAs Schottky contacts under a forward bias^24^,^33^,^34^. Yet we expect that the fundamental operation of our SV device is far more complicated than these cases with a single FM/SC interface. This is driven by the fact that our present two-terminal device exhibits two oppositely biased Schottky contacts, so that the subtle balance between their respective bias-dependent barrier profiles should in turn determine the overall performance of the device. To verify this hypothetic picture, we have investigated the bias dependence of the device MR at 5 K. As shown in Fig. 4(a), in the forward bias regime, the MR starts emerging at 0.21 V and increases with increasing forward bias within region (1), until reaching a maximum at 0.32 V, *i.e.* region (2). In region (3), the MR then decreases and eventually drops off sharply. The trend identified at region (1) is associated with an increased number of electrons tunneling into the GaAs membrane from the injector contact as the bias voltage increases. At region (2), where the maximum MR is observed, the energy bands of the Fe/GaAs Schottky contacts sustain an optimum condition for both spin injection and detection. While the MR decay at higher biases observed within region (3) might be due to the co-existence of electron tunneling and thermionic emission at the detector contact. It is important to point out that in the thermionic emission, where a portion of the injected electrons is sufficiently energetic to surmount the detector barrier height, the overall spin-dependent signal will be diluted, since these thermally transmitted electrons are not subjected to the spin-filtering at the detector interface^24^,^33^. For ease of clarification, the band alignments of the device under zero and biased conditions are schematically depicted in Fig. 4(b,c). On the contrary, even though the device MR under reverse bias shows a similar dependence as in forward bias, its maximum value is slightly lower and the voltage range for obtaining the MR becomes narrower. Here, we regard such a clear asymmetry in the observed bias-dependent MR as evidence of electrical spin injection and detection at the energetically different Fe/GaAs Schottky contacts in our two-terminal SV device.

### Theoretical analysis and discussions

To account for the clear but modest MR signals in the device, let us refer to the theoretical framework developed in ref. 22. Based on their one-dimensional flat-band model^35^, the authors have concluded that a significant MR response in their two-terminal FM/SC/FM device can only exist within a narrow window related to the contact resistance, or in the model convention, the interfacial resistance r_b_* of the FM/SC contacts^22^. In this work, the Fe/GaAs contact resistance is on the order of 10^−7^ Ωm^2^, which satisfies the lower edge of the window for spin injection, ρ_sc_*t_sc_ = 10^−13^ Ωm^2^ < r_b_*, where ρ_sc_ and t_sc_ are, respectively, the resistivity and thickness of the GaAs membrane. As for spin detection, r_b_* has to be lower than ρ_sc_*(l_sf_^sc^)^2^/t_sc_ = 10^−10^ Ωm^2^, where l_sf_^sc^ stands for the spin diffusion length in the GaAs. From this analysis, it is obvious that the corresponding r_b_* in our device is three orders of magnitude larger than the detection limit, thus explaining the modest MR value observed here in this work. Nevertheless, one may further correlate the corresponding MR to other decisive parameters, including the interfacial spin polarization γ and relevant time scale, such as the dwell time *τ*_*n*_ and the spin lifetime *τ*_*sf*_ of the electrons injected into the membrane.


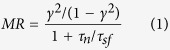


while γ and *τ*_*n*_/*τ*_*sf*_ cannot be both extracted from the measured MR via equation (1) 22, if, not rigorous though, we take γ = 0.16 as an upper bound value for the Fe/GaAs Schottky-tunnel type contact^4^, then 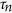/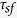 ≈ 4. Previous time-resolved Faraday rotation measurements on bulk *n*-GaAs yielded spin lifetimes close to 300 ps for *n* = 1 × 10^18^ cm^−3^ and less than 40 ps for *n* = 5 × 10^18^ cm^−3^ at 5 K^29^. If *τ*_*sf*_ is tentatively assumed to be 60 ps in our GaAs membrane, *τ*_*n*_ will be ~240 ps. Such a dwell-time-dominated electron transport has also been observed in spin-based transport devices based on *n*-doped Si^36^, and is here originated from the spatially inhomogeneous electric field distribution due to carrier depletion of the membrane sandwiched by the Schottky contacts. The resulting non-linear conduction band and potential minimum in the GaAs membrane may accommodate the injected electron spins for a time scale characterized by *τ*_*n*_, subsequently leading to depolarization of the spins before their arrival at the detector contact. Such a picture would sound even more plausible, if one considers the relatively low bias device operation and also close proximity of the interfacial depletion regions that are only few tens of nanometers apart at most.

The Fe/GaAs interfaces form a fundamental yet highly crucial part of the SV device, and their robustness has a direct impact on the resulting MR response. Due to the geometric constraints imposed by our specially designed MBE sample holders (see the Supporting Information), *in-situ* characterizations of the GaAs membrane as well as the deposited Fe electrodes are practically not possible. We are therefore unable to comment on crucial issues, such as the GaAs surface termination^37^,^38^, the Fe film crystallinity, and possible existence of “magnetic dead layers” at the Fe/GaAs interfaces^39^,^40^. However, we have taken an indirect approach to justify these interface effects by examining the device MR as functions of *in-situ* surface treatments on the GaAs membranes. As summarized in Table 1, both Ar^+^ ion sputtering and post-annealing are highly essential steps for successful observation of the MR effect. In addition, the exact temperature being utilized for the post-annealing has found to be very crucial. Since the GaAs surface reconstruction is known to depend very sensitively on the details of these treatments^37^,^38^,^39^,^41^, we believe that the membrane surface conditions, prior to the Fe depositions, should play a decisive role in determining the resulting electronic and magnetic properties of the FM/SC Schottky contacts, and consequently the performance of the two-terminal SV devices.

## Conclusions

We have demonstrated a spin injection and detection related MR effect in two-terminal Fe/GaAs(100)/Fe vertical spin-valves with the thickness of the GaAs layer down to 50 nm and the Fe pads deposited at the same growth run. The effect, which is sensitive to both temperature and bias, persists up to 20 K with a moderate value. The device operation is dwell-time dominated and is determined the subtle balance between the oppositely biased Fe/GaAs Schottky contacts. Last but not the least, the GaAs membrane surface conditions, prior to any Fe depositions, is found to be of critical importance, directly impacting the MR observation in the two-terminal devices. Our work has demonstrated the feasibility and the challenges in developing the vertical spin transistors, which has the potential for the high density 3D integration.

## Methods

### Device fabrication

The GaAs(100) wafers used in this study consist of epilayers of Al_0.7_Ga_0.3_As and GaAs with the following structure: amorphous As-cap/GaAs (50 nm, *n*-type, 3 × 10^18^ cm^−3^)/Al_0.7_Ga_0.3_As (500 nm, undoped)/500 μm thick semi-insulating (S.I.) GaAs(100). The samples were mounted upside down on dummy Si wafers by photoresist and thinned down to about 100 μm using H_2_SO_4_-based etchant. Optical lithography was performed on the thinned samples from the backside to prepare 100 μm square mesa. Selective chemical etching was done through the mesa using a mixture of NH_4_OH and H_2_O_2_, which selectively etches GaAs and stops on the AlGaAs epilayer. The AlGaAs etch-stop layer was further removed by diluted HF. The samples were then detached from the Si wafer and cleaned in acetone and IPA thoroughly prior loading into an UHV-MBE chamber with a base pressure of 2 × 10^−10^ mbar. While in the chamber, the etched membranes surface was sputter-cleaned by low energy Ar^+^ ion at RT, followed by annealing at 773 K for 1 h. Subsequent growth of Fe films by electron-beam evaporation was done at a chamber pressure better than 1 × 10^−9^ mbar at RT through a shallow mask (see the Supplementary Information for details). The Fe layers were capped with 20 ML of Cr to avoid oxidation during *ex-situ* analysis.

### Focused MOKE

The high resolution focused MOKE magnetometer consists of a stabilized 5 mW continuous HeNe laser of 635 nm wavelength, a stack of focusing lenses and a nano-positioning stage. The focused laser spot size was measured to be less than 1 μm and the sensitivity allows for measurements of a 10 ML Fe film in single shot loops.

### Transport measurement

For electrical measurements, the devices were covered by thermally evaporated 400 nm thick Au on both sides and attached upside down to a multipin chip. I–V and MR measurements were performed in the CPP geometry at various temperatures from 300 down to 5 K.

## Additional Information

**How to cite this article**: Wong, P. K. J. *et al*. Spin-Dependent Transport in Fe/GaAs(100)/Fe Vertical Spin-Valves. *Sci. Rep.* **6**, 29845; doi: 10.1038/srep29845 (2016).

## Supplementary Material

Supplementary Information

## Figures and Tables

**Table 1:** *In-situ* surface treatments on etched GaAs membranes, prior to MBE growth of Fe electrodes, and their relations to device MR.

*In-situ* surface treatments	MR signal
Annealing at 673 K	No
Annealing at 773 K	No
Ar^+^ sputtering + post-annealing at 673 K	No
Ar^+^ Sputtering + post-annealing at 773 K	Yes
