# Supplementary material for: Spin-Dependent Transport in Fe/GaAs(100)/Fe Vertical Spin-Valves
Source: Sci Rep. 2016 Jul 19;6:29845. doi: 10.1038/srep29845 (PMC4949422; doi:10.1038/srep29845)
Supplement: Supplementary Information [file srep29845-s1.pdf]

Supplementary Information

Spin-Dependent Transport in Fe/GaAs(100)/Fe Vertical Spin-Valves

P. K. Johnny Wong[1,2†], Wen Zhang[2,†], Jing Wu[3], Iain G. Will[2], Yongbing Xu[1,2*], Ke Xia[4], Stuart N. Holmes[5], Ian Farrer[6], Harvey E Beere[7] and David A. Ritchie[7]

[1] *York-Nanjing Joint Center in Spintronics and NanoEngineering, School of Electronics Science and Engineering, Nanjing University, Nanjing 210093, China*

[2]*Spintronics and Nanodevice Laboratory, Department of Electronics, University of York, YO10 5DD, UK*

[3]*Department of Physics, University of York, YO10 5DD, UK*

[4]*Department of Physics, Beijing Normal University, Beijing, China*

[5] *Toshiba Research Europe Limited, Cambridge Research Laboratory, 208 Cambridge Science Park, Milton Road, Cambridge, CB4 0GZ, UK*

[6] *Department of Electronic & Electrical Engineering, The University of Sheffield, Mappin Street, Sheffield S1 3JD*

[7]*Cavendish Laboratory, University of Cambridge, Madingley Road, Cambridge, CB3 0HE, UK*

†*Present addresses: NanoElectronics Group, MESA+ Institute for Nanotechnology, P.O. Box 217, University of Twente, 7500 AE, Enschede, The Netherlands (P.K.J.W.); Department of Physics, National University of Singapore, 2 Science Drive 3, 117542, Singapore (W.Z.); Department of Electronic and Electrical Engineering, University of Sheffield, Mappin Street, Sheffield, S1 3JD, UK (I.F.).*

Correspondence and requests for materials should be addressed to Y.B.X. (email: yongbing.xu@york.ac.uk)

Figure S1a

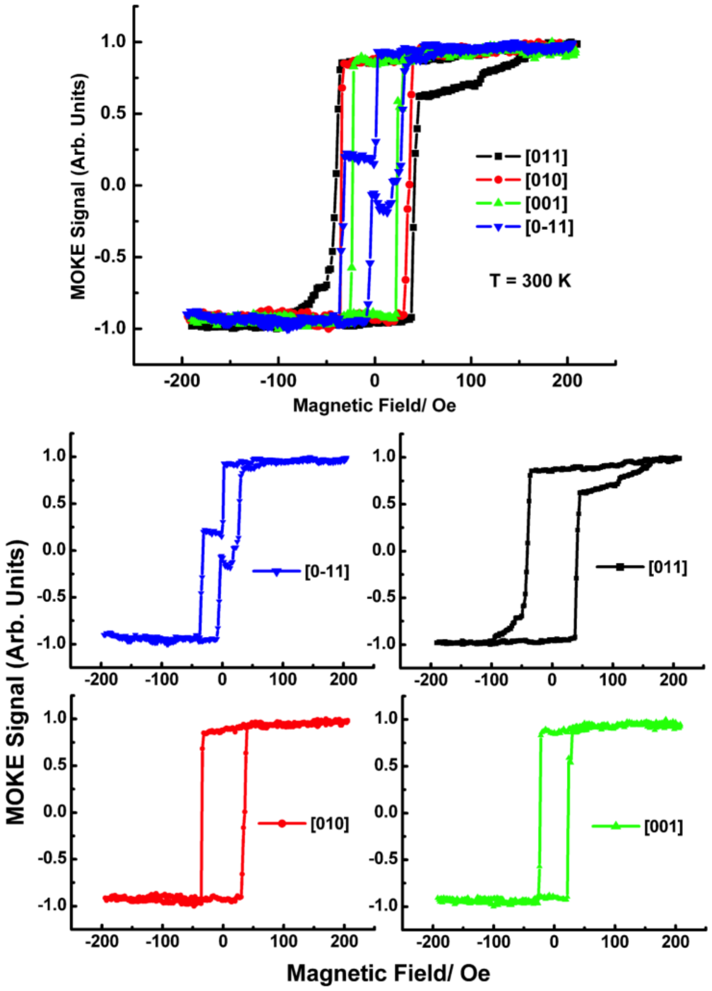

Figure S1a| RT longitudinal magnetic hysteresis loops of the 150 ML Fe electrode along different applied field azimuths with respect to the four major crystallographic axes of the GaAs(100) membrane.

Figure S1b

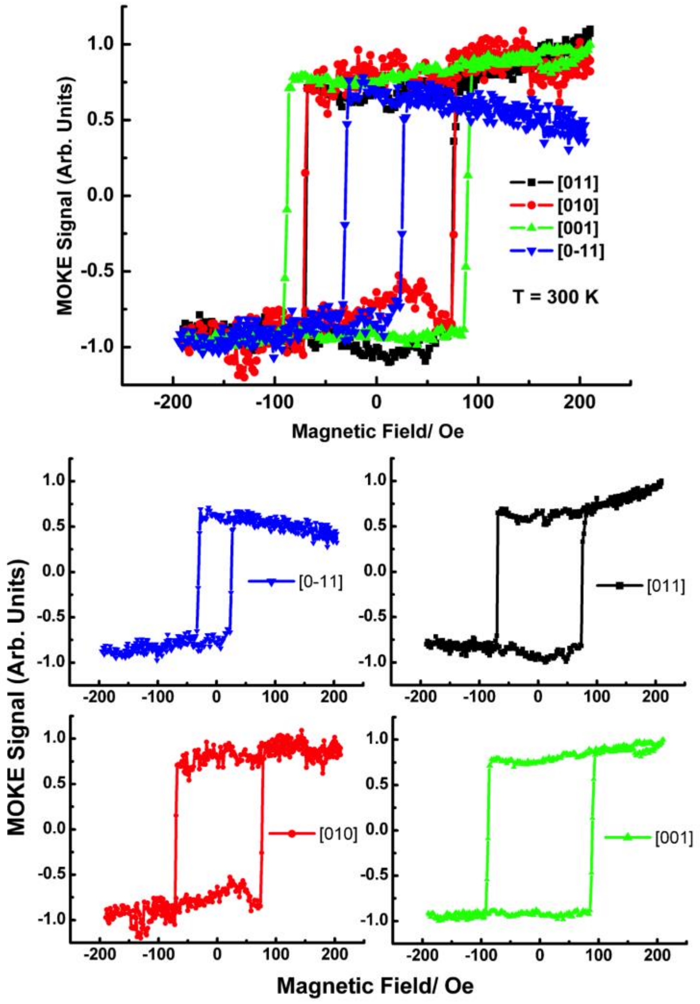

Figure S1b| RT longitudinal magnetic hysteresis loops of the 10 ML Fe electrode along different applied field azimuths with respect to the four major crystallographic axes of the GaAs(100) membrane.

Figure S2

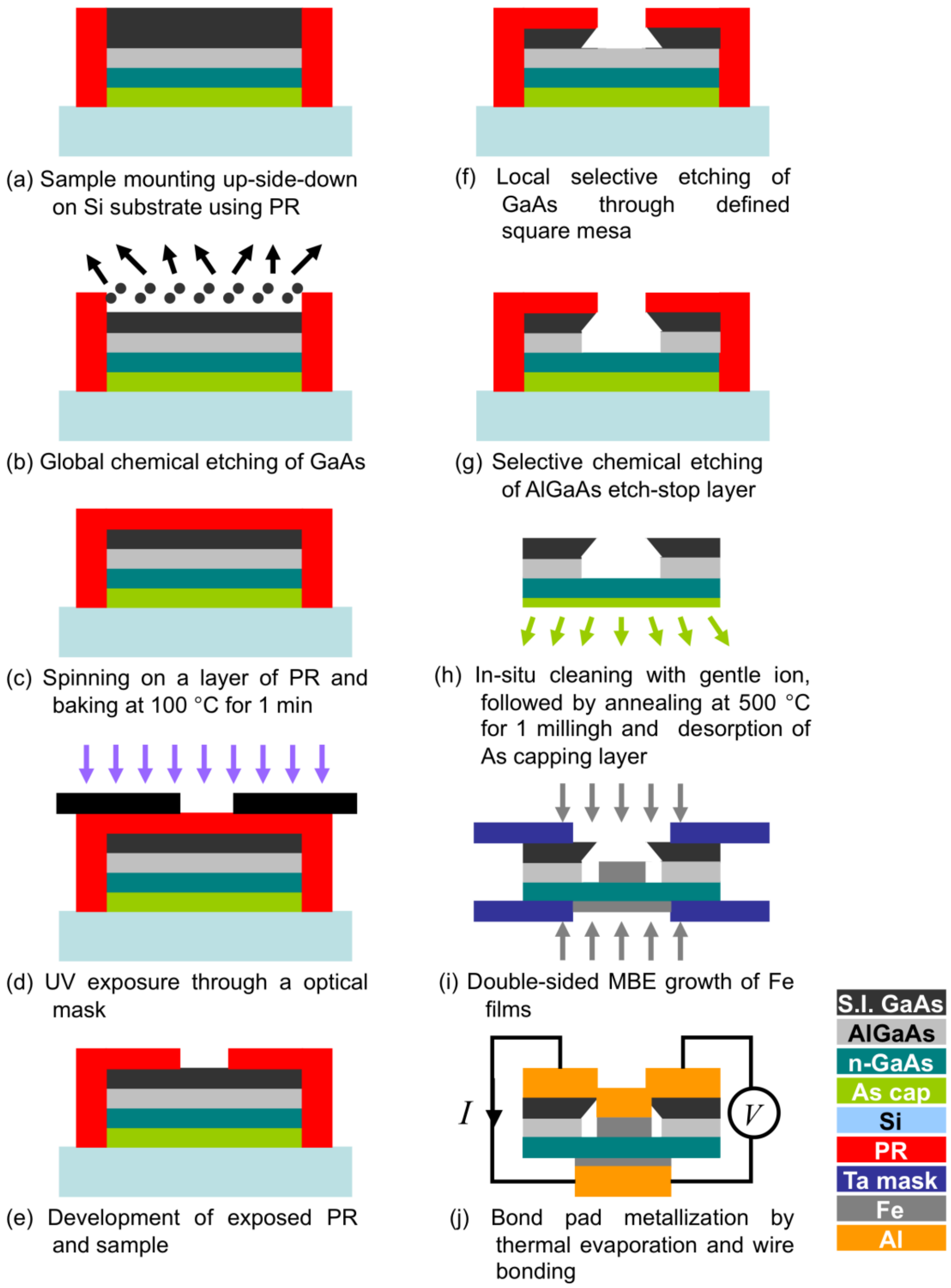

Figure S2| Flow diagram of Fe/GaAs(100)/Fe vertical SV device fabrication.

MBE sample holder for double-sided growth of Fe electrodes on GaAs membrane

In-situ growth of Fe electrodes on both sides of the GaAs membrane without breaking a vacuum have been achieved by using a home-made sample holder as shown in Fig. S3. This holder, which is made of tantalum (Ta), has been designed to have three separate layers, and the whole assembly can be flipped from one side to the other using a wobble stick. The top and bottom layers are each consisting of six circular holes with an identical diameter of 1.5 mm. These plates sandwich the middle layer that has with six open rectangular grooves (2.5×3.5 mm^2) within which the etched GaAs membranes can be rigidly accommodated. The thicknesses of all these layers have been chosen to be 125 μm, which are sufficiently thin but at the same time hard enough to withstand high heating and sample transfer processes *in-situ*.

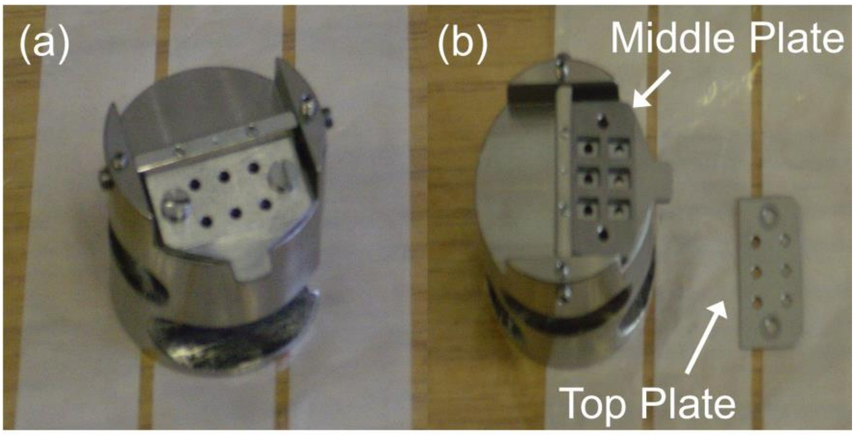

Figure S3| Diagrams of the UHV MBE sample holder for double-sided growth of Fe/GaAs(100)/Fe vertical SV devices.
